# Assessing the Risk of Hypertension in Chronic, Elderly Patients during the COVID-19 Pandemic: A Prospective Study

**DOI:** 10.3390/jcdd11010021

**Published:** 2024-01-12

**Authors:** Miguel Quesada-Caballero, Ana Carmona-García, Rubén A. García-Lara, Antonio M. Caballero-Mateos, Nora Suleiman-Martos, Guillermo A. Cañadas-De la Fuente, José L. Romero-Béjar

**Affiliations:** 1 Centro de Salud Albayda La Cruz, Distrito Sanitario Granada-Metropolitano, Servicio Andaluz de Salud, Calle Virgen de la Consolación, 12, 18015 Granada, Spain; miguel.quesada.caballero.sspa@juntadeandalucia.es; 2 Critical Care and Emergency Unit (UCCU), Distrito Sanitario Granada-Metropolitano, Servicio Andaluz de Salud, Calle Virgen de la Consolación, 12, 18015 Granada, Spain; ana.carmona.sspa@juntadeandalucia.es; 3 Íllora Health Center, Granada-Metropolitan Health District, Andalusian Health Service, Calle Virgen de la Consolación 12, 18015 Granada, Spain; ruben.garcia.lara.sspa@juntadeandalucia.es; 4 Instituto de Investigación Biosanitaria (ibs.Granada), 18012 Granada, Spain; jlrbejar@ugr.es; 5 Gastroenterology and Hepatology Department, San Cecilio University Hospital, Av. del Conocimiento s/n, 18016 Granada, Spain; 6 Faculty of Health Sciences, University of Granada, 18016 Granada, Spain; norasm@ugr.es (N.S.-M.); gacf@ugr.es (G.A.C.-D.l.F.); 7 Brain, Mind and Behaviour Research Center (CIMCYC), University of Granada, 18071 Granada, Spain; 8 Statistics and Operational Research Department, University of Granada, Avda. Fuentenueva S/N, 18071 Granada, Spain; 9 Institute of Mathematics, University of Granada (IMAG), Ventanilla 11, 18001 Granada, Spain

**Keywords:** older, hypertension, SARS-CoV-2 infection, hypercholesterolemia, COVID-19 disease, cardiovascular disease

## Abstract

Background: This study considers care management for older chronic patients during and after the COVID-19 pandemic. Aims: To identify groups of variables at previous time points as a basis for deriving efficient classification models during and after a pandemic situation and to quantify the effect of each variable within the model to predict levels of worsening risk in diastolic and systolic arterial hypertension (AHT). Material and Methods: In this prospective longitudinal study, data were collected at three time points: before, during, and after the COVID-19 pandemic period. Results: The study included 148 patients with an average age of 81.6 years. During the study period, mean systolic blood pressure among this population rose by 5 mmHg to 128.8 mmHg; the number of patients with systolic blood pressure > 140 mmHg rose by 45.3%; among those with diastolic blood pressure > 90, the number rose by 41.2%; mean triglycerides levels rose to 152.6 mg/dL; cholesterol levels rose to 147 mg/dL; and LDL cholesterol rose to 112.2 mg/dL. Meanwhile, mean levels of HDL cholesterol decreased to 46.5 mg/dL. Binary-response logistic regression models were constructed to identify the most relevant variables for predicting AHT risk during and after the pandemic. The heart rate (OR = 1.79; 95% CI: 1.22–2.72) and body mass index (OR = 1.75; 95% CI: 1.08–2.94) variables were significant at the population level (*p* < 0.05) for diastolic and systolic AHT in the pandemic period risk models. The body mass index variable was also significant for diastolic AHT in the post-pandemic period risk model (OR = 1.97; 95% CI: 1.32–2.94), whilst the triglycerides variable was significant in the systolic AHT post-pandemic period risk model (OR = 1.49; 95% CI: 1.01–1.86). Conclusions: Bad control of arterial hypertension in older patients with chronic disease is associated with elevated levels of LDL cholesterol, total cholesterol, systolic blood pressure, heart rate and triglycerides, and lower levels of HDL cholesterol.

## 1. Introduction

Cardiovascular risk is the probability of a pathological cardiovascular event occurring in a given time period. It is defined by the cardiovascular risk factors of patients belonging to a given social group [1]. The main factors leading to heart disease are diabetes, arterial hypertension (AHT), fatty diet, sedentary lifestyle, smoking, obesity, overweight, and high cholesterol. However, with appropriate awareness, all of these factors can be addressed, and the impact of cardiovascular diseases can be reduced [2]. The main treatment for chronic hypertensive patients is the use of angiotensin II receptor blockers [3]. These drugs are safe and have been recommended in the context of COVID-19 [4]. Heart disease currently accounts for 16% of all deaths from all causes [5,6], and arterial hypertension (AHT) remains the most prevalent cause of cardiovascular morbidity and mortality [7]. However, studies have reported that reducing systolic and diastolic blood pressure in hypertensive patients by 5 mmHg reduces cardiovascular events by 10%, cerebrovascular events by 15%, and mortality by 5% [8,9].

The prevalence of AHT has risen steadily, mainly as a result of the aging of the population. The size of the population aged over 80 years has grown exponentially over the past 40 years. In older adults, arterial stiffness is the major cause of elevated systolic blood pressure (SBP), reduced diastolic blood pressure (DBP), and elevated pulse pressure (PP). The latter value is calculated as SBP minus DBP. These age-related alterations in BP are powerful determinants of major cardiovascular disease (CVD) events and of all-cause mortality. Both SBP and DBP increase with age up to the age of 50–60 years. Over this age, in most cases, SBP increases with age, whereas DBP remains stable or even decreases spontaneously [10]. The lifetime risk of developing hypertension exceeds 90% if the person lives long enough. In the Hypertension in the Very Elderly Trial (HYVET), a significant reduction in mortality, fatal strokes, and heart failure was observed in the more intensively treated group (BP target < 150 mm Hg). Moreover, the Berlin Initiative Study of patients aged 70 years and older treated with antihypertensive medication at baseline reported that BP values < 140/90 mm Hg may be associated with an increased mortality risk in older patients and in those with previous CV events. Overall, but particularly in older patients, the authors of this study highlighted the benefits of employing an individualized BP approach in the context of frailty and tolerability, two factors of crucial importance in determining the benefits and possible harm produced by lowering BP [7]. In fact, these are the main causes of death and disability worldwide. The early diagnosis and control of chronic disease, including AHT, prevents complications and enhances the quality and duration of life [11]. 

Dyslipidaemia, abdominal obesity, and chronic inflammation are also known risk factors for cardiovascular disease. Dyslipidaemia provokes endothelial damage, resulting in a loss of vasomotor activity, which could then lead to hypertension. Moreover, there is evidence of a relationship between increased weight and increased BP. Similarly, elevated BP is reported to be associated with inflammation in the general population; indeed, even at the low levels of inflammation typically observed in the general population, there is an association between the level of C-reactive protein, as a marker of inflammation, and SBP [12].

By August 2023, the coronavirus SARS-CoV-2 had caused 768 million cases and 6.9 million deaths worldwide [1]. The most common comorbidity of COVID-19 is hypertension (present in 31.2% of those infected with the virus and in 58.3% of those with severe COVID-19) [13]. In response to the pandemic, governments worldwide imposed lockdowns and restrictions on mobility, seeking to prevent contagion. From the outset, social distancing was one of the main measures used to prevent contagion. However, this measure made it difficult to control cardiovascular risk factors in heart patients [14,15]. Telemedicine offers a useful alternative to face-to-face consultations, but the patients must be familiar with this technology, and older chronic patients often have difficulty in this respect [16]. During the COVID-19 pandemic, the frequency of face-to-face medical encounters to prevent infection decreased as a result of lockdowns and other restrictions [13]. This situation made it more difficult to control and manage the condition of chronic patients, heightened the risk of undesirable events, and exacerbated mental disorders such as anxiety and depression [17,18].

In view of these considerations, it would be useful to construct an explanatory model using sociodemographic and clinical variables to estimate the risk of worsening SBP and DBP among elderly patients with chronic disease from one time point to another during a pandemic situation. In this estimation, the model should also provide a measure of the strength of these variables. Accordingly, the aim of the present study is to consider appropriate sociodemographic and clinical variables, measured at consecutive time points, to obtain an efficient classification model of SBP and DBP risk during and after a pandemic situation. We then quantify the effect on SBP and DBP of each variable within the model.2. 

## 2. Materials and Methods

### 2.1. Design

In this prospective longitudinal study, data were collected at three time points: before, during, and after the COVID-19 pandemic. The study participants were assessed at these time points, and data on sociodemographic and clinical variables of interest were recorded.

### 2.2. Participants

The study participants were 148 patients recruited from the Metropolitan Health District of Granada (Spain) via incidental sampling in the Primary Care consultation. Patients were recruited and gave consent between December 2021 and February 2022. At this time, the first measure (T1) was taken. Then, after approximately seven months (September 2022 to December 2022), the second measurement (T2) was taken. The average age of the participants was 81.6 years (SD = 9.65), and 66.2% were female.

### 2.3. Eligibility Criteria and Procedure

The following inclusion criteria were applied: (1) patients with a diagnosis of chronic AHT, regardless of other pathologies; (2) over 65 years of age; (3) regular follow-up by primary care professionals; (4) informed agreement to participate in the study. 

The exclusion criteria were: (1) patients who have been displaced, were not followed up, or were assigned to the Granada-Metropolitan Health District during the last three years; (2) refusal to participate in the study.

The variables analyzed were collected in the consultation room in person. Safety measures and social distance from these patients were respected at all times.

### 2.4. Statistical Analysis

First, a graphical exploratory analysis was conducted to investigate individual, paired, and triplet variables with potential predictive power. Second, binary-response logistic regression models [19] were employed to identify the most relevant variables for predicting AHT risk (both systolic and diastolic). A stepwise forward–backward selection model was chosen without considering interactions, as this best suited the data. The goodness-of-fit of the models was assessed using the probability ratio test and Stukel’s chi-squared test. Wald’s test was applied to evaluate the significance of factors in the models at the population level. The model’s validity was confirmed by calculating the rate of correct classifications. Additionally, the performance of the models was analyzed using the ROC curve. The strength ratios for each level relative to the adjacent level were determined, considering potential variations in the risk sub-scales examined. The statistical analysis was performed using R Statistical Computing Software (version 4.1.1).

### 2.5. Ethical Considerations

This study was carried out in accordance with the 1975 Declaration of Helsinki [20] and was approved by the Clinical Research Ethics Committee of the Andalusian Public Health System (TES-COVID-RGL).

## 3. Results

This section is organized as follows. First, we present a descriptive analysis of the response and explanatory variables considered. Then, we conduct an exploratory analysis using graphical outputs to assess the classification power of individual and grouped variables in predicting the risk for high values of AHT (both diastolic and systolic). In Section 3.3, a binary logistic regression model is constructed to estimate the risk of high AHT levels according to the sociodemographic and clinical variables collected at each time point. A comprehensive examination is then made of the prognostic power of each variable. Additionally, various measures and graphical representations are provided to evaluate the quality of the model in terms of inferential power, accuracy, and validity.

### 3.1. Sample Description

The pre-pandemic, pandemic, and post-pandemic periods are denoted by T1, T2, and T3, respectively. Table 1 shows a descriptive analysis of the clinical and sociodemographic variables considered at T1, T2, and T3. For the classification models, the variables AHT_SBP and AHT_DBP were segmented and coded. Table 1 also summarizes the coding system used for these variables at each time point.

### 3.2. Exploratory Analysis for Classification

#### 3.2.1. Univariate Graphical Exploratory Analysis

**Figure 1:**
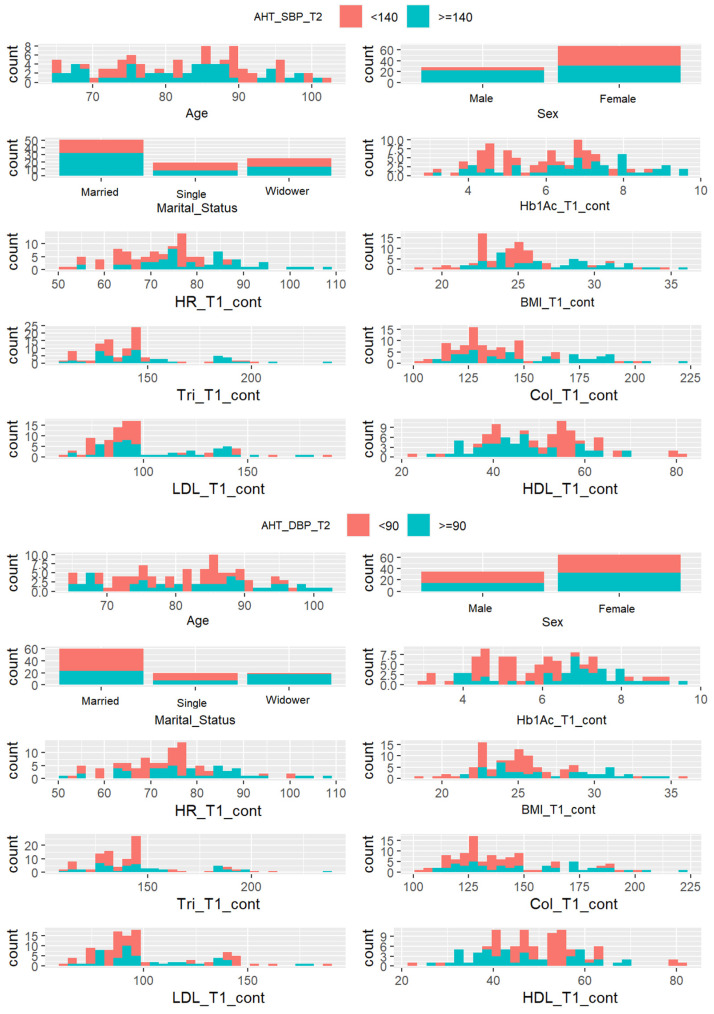
Systolic AHT (**top**) and diastolic AHT (**bottom**) classification based on individual variables.

Figure 1 shows the overlapping histograms used for the classification of the risk of presenting high levels of systolic and diastolic AHT based on age, sex, and each clinical variable considered independently. Sex (male) and high values of HR, BMI, Tri, Chol, and LDL variables seem to be adequate as independent classifiers for systolic AHT. This is reflected in their histograms in Figure 1 (top) because the blue color that corresponds to a high level of systolic AHT is well separated for high values of these variables. On the other hand, high values of HR and BMI seem to be adequate for diastolic AHT, as shown in Figure 1 (bottom).

#### 3.2.2. Bivariate Graphical Exploratory Analysis

**Figure 2:**
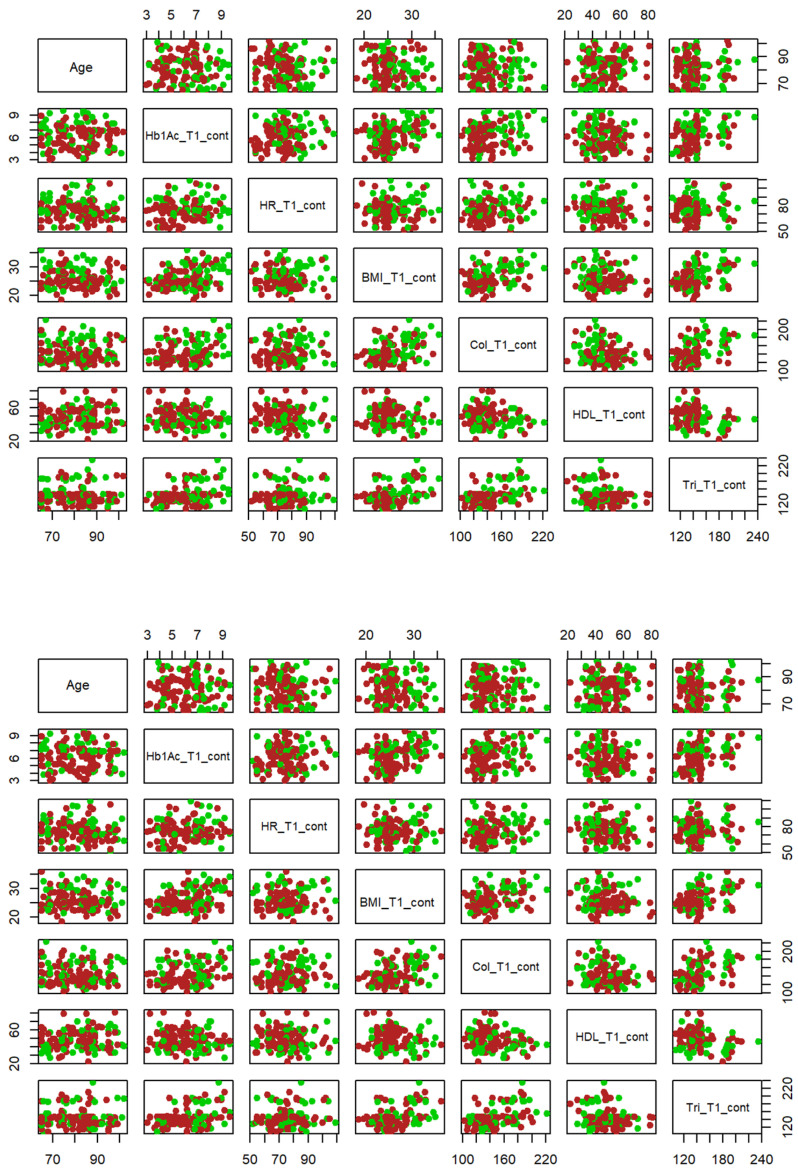
Systolic AHT (**top**) and diastolic AHT (**bottom**) classification based on two variables. Note: Hb1Ac = glycosylated hemoglobin, pressure, HR = heart rate, Tri = triglycerides, Chol = cholesterol, HDL = high-density lipoprotein, BMI = body mass index. Green dots indicate AHT >= 140 whilst red dots indicate AHT < 140.

Figure 2 shows the potential applicability of classifiers of each pair of variables. The BMI and cholesterol (Chol) variables are jointly adequate classifiers for systolic AHT because the green and red dots are well separated in the corresponding bi-plot located (according to a matrix notation) in the fourth row with the fifth column. However, this pair of variables does not seem to be adequate for classifying diastolic HTA.

#### 3.2.3. Three-Subscale Graphical Exploratory Analysis

**Figure 3:**
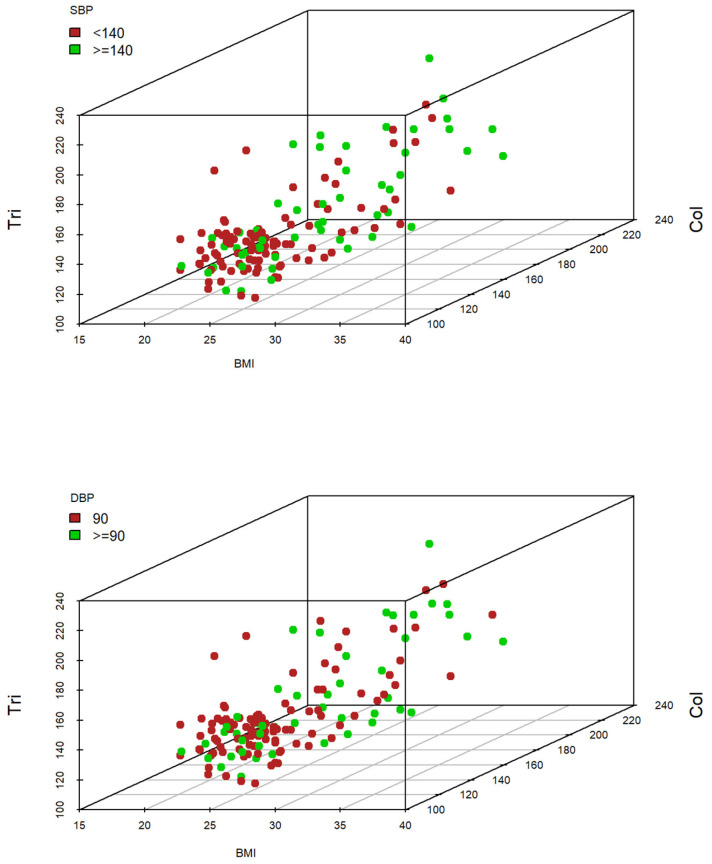
Systolic blood pressure (SBP) (**top**) and diastolic blood pressure (DBP) (**bottom**) classification based on three variables.

The cholesterol (Chol) variable, jointly with triglycerides (Tri) and BMI, provides adequate classifiers both for systolic and diastolic ATH, as shown by 3D scatterplots in Figure 3 since high values of these variables are identified simultaneously with high values of ATH (green dots).

### 3.3. Logit Model to Predict Systolic and Diastolic AHT Level at T2 Based on Sociodemographic and Clinical Variables at T1

This section presents a binary-response logistic regression model used for classifying systolic AHT levels according to age and sex and to the clinical variables measured at T1. The stepwise forward–backward selection model included the variables sex, HR, BMI, and cholesterol in the binary logistic regression model, as relevant for the prognosis of systolic AHT. 

The model used to predict systolic AHT level is

$${\hat{L}}_{i}\left(hr,imc,col\right)={\hat{B}}_{0}+{\hat{B}}_{Sex}{\left(Sex\right)}_{i}+{\hat{B}}_{HR}hr+{\hat{B}}_{IMC}imc+{\hat{B}}_{Col}col;\;i=0,\;1;\;{\left(Sex\right)}_{0}=0$$

While that for diastolic AHT is

$$\hat{L}(hr,imc)={\hat{B}}_{0}+{\hat{B}}_{HR}hr+{\hat{B}}_{IMC}imc$$

The parameters estimated for each subscale in the binary logistic regression models are shown Table 2.

The chi-square log-likelihood test statistic for the diastolic AHT level prediction model is X^2^(8, N = 148) = 163.12, *p* < 0.001. When these variables are included in the model, the fit improves significantly compared to a model that only takes the constant into account. The result of the Stukel goodness-of-fit test for this model was X^2^(2, N = 148) = 5.697, *p* = 0.058. These results show that the model produces a good fit at the population level for a worsening risk of systolic AHT.

The results of the z-test (see Table 2) show that HR and BMI are significant at a population-based level (*p* < 0.05). The change ratio prognosis for the levels considered (no risk vs. risk of increasing level of systolic AHT) was calculated for all the significant explanatory variables. For HR, for example, the chances of high systolic AHT for a ten-unit increment in this variable (unit increments are not illustrative or relevant) are almost twice as great (OR =1.79; 95% CI: 1.22–2.72), while for BMI, the odds of high systolic AHT are almost twice as great among patients in whom this index increases by four units (OR = 1.75; 95% CI: 1.08–2.94). 

Finally, this model has a 77.0% correct classification rate and an area under the ROC of 75.29%, which attests to the good discrimination ability of the model for systolic AHT. The following values were obtained for the parameters of internal validity and predictive value: sensitivity = 76.99%; specificity = 77.14%; positive predictive value = 91.58%; negative predictive value = 51.92%.

For the systolic AHT level prediction model, the chi-square log-likelihood test result was X^2^(8, N = 148) = 173.314, *p* < 0.001. Therefore, when these variables are included in the model, the fit improves significantly compared to a model that only takes the constant into account. The Stukel goodness-of-fit test for this model was X^2^(2, N = 148) = 0.28, *p* = 0.869 > 0.05. These results show that the model produces a good fit at the population level for the diastolic AHT level prediction.

Once again, as seen in the results of the z-test (see Table 2), only BMI is significant at a population-based level (*p* < 0.05). In this case, the prognosis ratio for high diastolic AHT is almost twice as great among patients for whom this index rises by 4 units (OR = 1.89; 95% CI: 1.25–2.91).

In line with the previous model, this model has a correct classification rate of 72.9% and an area under the ROC curve of 66.65%. Its sensitivity and specificity are 73.43% and 70.00%, respectively, and the positive and negative predictive values are 94.00% and 29.16%, respectively.

### 3.4. Logit Model to Predict Systolic and Diastolic AHT Level at T3 Based on Sociodemographic and Clinical Variables at T2

The estimated model for systolic AHT risk level prediction at T3 based on the sociodemographic variables and clinical variables obtained at T2 is

$$\hat{L}(hr,imc,tri)={\hat{B}}_{0}+{\hat{B}}_{HR}hr+{\hat{B}}_{IMC}imc+{\hat{B}}_{Tri}tri$$

And that for diastolic AHT at T3 is

$$\hat{L}(imc)={\hat{B}}_{0}+{\hat{B}}_{IMC}imc$$

Table 3 includes the parameters considered for each model.

The chi-square log-likelihood test result for the systolic AHT model was X^2^(8, N = 148) = 183.74, *p* < 0.001. When these variables are included in the model, the fit improves significantly compared to a model that only takes the constant into account. The Stukel goodness-of-fit test result for this model was X^2^(2, N = 148) = 0.19, *p* = 0.91. In view of these results, we conclude that the model produces a good fit at the population level for the risk of increasing systolic AHT at T3. 

Once again, according to the results of the z-test (see Table 3), the only significant variable was that of triglycerides (*p* < 0.05). Thus, if a patient’s level of triglycerides rose by 20 units, the possibility of higher levels of systolic AHT would almost double (OR = 1.49; 95% CI: 1.01–1.86).

In line with the previous cases, this model has a correct classification rate of 67.6% and an area under the ROC of 69.17%. The following values were obtained for the internal validity parameters and the safety indices: sensitivity = 67.67%; specificity = 70.83%; positive predictive value = 82.71%; negative predictive value = 50.74%.

The chi-square log-likelihood test result for the prognosis model for diastolic AHT was X^2^(8, N = 148) = 188.77, *p* < 0.001. When these variables are included in the model, the fit improves significantly compared to a model that only takes the constant into account. The Stukel goodness-of-fit test result for this model was X^2^(2, N = 148) = 0.55, *p* = 0.76. These results show that the model produces a good fit at the population level for the risk of increasing systolic AHT at T3. 

According to the z-test results (see Table 3), only BMI was a significant variable (*p* < 0.05). Among patients whose BMI rises by 4 units, the possibility of higher levels of diastolic AHT almost doubles (OR = 1.97; 95% CI: 1.32–2.94).

This model has a correct classification rate of 63.5% and an area under the ROC of 69.17%. 

The following values were obtained for the internal validity parameters and the safety indices: sensitivity = 65.13%; specificity = 58.97%; positive predictive value = 81.61%; negative predictive value = 37.70%.

## 4. Discussion

In this study, we seek to identify groups of variables that constitute a basis for obtaining efficient models to classify and quantify the risk of diastolic and systolic AHT during and after a pandemic situation. Four models were obtained, providing a first approximation of changes in the risk of high levels of systolic and diastolic blood pressure. Two of these models refer to systolic and diastolic AHT during the pandemic situation, based on clinical markers in the pre-pandemic period. The other two models concern systolic and diastolic AHT at a post-pandemic time point based on clinical markers in the pandemic situation. These models include the following variables: body mass index, cholesterol, triglycerides, and female sex.

The rising levels of AHT recorded during and after the pandemic may reflect the more sedentary lifestyles imposed by lockdowns and other restrictions. When physical activity is performed regularly, it alleviates CVD risk factors. However, recommendations should take into account individual particularities and be based on the principle that even a little is better than nothing. Physical activity can take many forms, including actions performed at work, in active forms of transport, performing household chores, or for recreation/sport. Physical exercise is a specific type of physical activity that is planned, structured, repetitive, and performed in order to improve or maintain physical fitness. Such activities are usually more beneficial when performed in moderation rather than in an intensive, concentrated manner [21,22].

Sedentary behavior is associated with an increased risk of cardiovascular events, elevated blood pressure and heart rate, a greater risk of cancer, and, in general, an increase in all causes of death [23,24,25]. Physical activity is known to protect against cardiovascular disease and to increase the quality of life, and almost any level of activity is better than sedentary behavior. Thus, for the general population, experts recommend moderate-intensive exercise for 150 min for 3–5 days a week. For the elderly population, this physical activity should be limited to 75 min of vigorous activity per week (such as jogging and running), at least 2 days a week [26,27].

During the pandemic-era lockdown, shopping opportunities were restricted, making it more difficult to consume a healthy diet. This factor may have contributed to the worsening health status results observed in the study population. Data from large cohort studies and the randomized clinical trial PREDIMED indicate that adherence to dietary patterns such as the Mediterranean diet confers evident cardiovascular benefits. By contrast, low-fat diets are currently being questioned as having little potential for cardiovascular protection. With regard to edible fats, virgin olive oil is the most effective culinary fat in the prevention of CVD. Nutritional interventions for a five-year period in the PREDIMED study showed that participants who consumed the Mediterranean diet supplemented with extra virgin olive oil or nuts experienced an average 30% reduction in major cardiovascular events, in addition to other beneficial effects such as a reduced risk of DM2 and atrial fibrillation. Eating fish or seafood at least three times a week, two of them in the form of oily fish, reduces CVD. With regard to dairy products, at least two servings per day should be consumed. Legumes and whole grain cereals contain many healthy nutrients, and their regular consumption is associated with a reduced risk of CVD. To promote cardiovascular health and reduce cholesterolemia, it is recommended that a serving of pulses should be consumed at least four times a week. Excessive salt consumption is associated with CVD and morbidity from cardiometabolic causes, and therefore, a low-salt diet (<5 g/day) is recommended [28].

Health accessibility was reduced in the pandemic era, and health control in hypertensive patients (blood test, heart rate evaluation, and hypertension evaluation) was decreased in comparison with pre-pandemic times [29]. This situation improves the unadjusted treatment, and professionals cannot apply the guidelines properly. Actually, the recommendations are to use a pharmacological treatment for all hypertensive patients except ATH type 1 with low cardiovascular risk. The objective is to attain arterial tension under 140/90 and optionally 130/80 in 18–65 years. The pharmacological treatment has 5 mean groups that have demonstrated reduced mortality and mobility: 1—ACEI (angiotensin-converting enzyme inhibitors), 2—ARAII (angiotensin II receptor antagonist), 3—Betablockers, 4—CA (calcium-antagonist), 5—Diuretics (Thiazides preferably or “Tiazides like” as indapamide). The basic strategy is to start with IECA or ARAII + Diuretics in low doses and evaluate in 15–30 days. If there is no control, increase to high doses and again re-evaluate in 15–30 days. Then, if there is no control, add one diuretic and re-evaluate in 15–30 days. Finally, if there is no control yet, add spironolactone 25–50 mg to reach optimal arterial tension [16]. Telehealth and home blood pressure monitoring have been shown to improve control in patients in the pandemic era, with good patient satisfaction. In fact, use by the elderly should be a priority, as control in the home is essential, but because they are unfamiliar with such devices, the devices are more of a hindrance than a help to them [16,30].

Among the possible limitations, longer follow-up of patients is needed to know the extent of complications in this age group. In addition, due to the strict measures of social distance by COVID-19, patient selection was complex. Patients were recruited by incidental sampling from a Primary Health Care Center when they came to the clinic in person. Furthermore, it would have been interesting to compare our parameters with other potential worsening factors, such as sedentary lifestyle, diet, and other parameters, such as glycosylated hemoglobin or body mass index, among others. The sample is, therefore, not fully representative, and the results should be used with caution.

In view of the restrictions imposed to address the COVID-19 pandemic and the negative consequences on health, appropriate guidelines and protocols should be created to optimize the use of telemedicine for chronic patients to manage their health care more efficiently. Furthermore, additional randomized clinical trials should be conducted to consider in greater detail how telemedicine might most effectively be used.

## 5. Conclusions

Inadequate control of arterial hypertension in older patients with chronic disease (as commonly occurred during the COVID-19 pandemic) is associated with elevated levels of LDL cholesterol, total cholesterol, systolic blood pressure, heart rate, and triglycerides, and lower levels of HDL cholesterol.

For this reason, international scientific societies have published clinical guidelines and statements advising careful control of cardiovascular risks in older chronic patients and the periodic reevaluation and optimization of treatment regimens.

## Figures and Tables

**Table 1:** Clinical and sociodemographic variables at T1, T2, and T3.

Descriptive Analysis of Sociodemographic Information				
Variable	Levels			% (N)
Sex (*N* = 148)	(0) Male			33.8 (50)
	(1) Female			66.2 (98)
Marital Status (*N* = 148)	(0) Married			56.1 (83)
	(1) Single			18.2 (27)
	(2) Widowed			25.7 (38)
Variable	Mean (SD)			
Age (*N* = 148)	81.6 (9.65)			
**Descriptive Analysis of Clinical Information**				
**Variable**	**Mean (SD)-T1**		**Mean (SD)-T2**	**Mean (SD)-T3**
Hb1Ac (*N* = 148)	6.2 (1.54)		6.2 (1.60)	6.2 (1.64)
AHT_SBP (*N* = 148)	123.7 (17.42)		127.3 (15.51)	128.8 (18.33)
AHT_DBP (*N* = 148)	77.7 (10.41)		79.5 (11.83)	77.7 (10.41)
HR (*N* = 148)	75.0 (11.35)		75.4 (10.55)	75.5 (11.09)
BMI (*N* = 148)	25.8 (3.44)		25.8 (3.51)	25.7 (3.56)
Tri (*N* = 148)	144.2 (23.16)		148.7 (28.68)	152.6 (26.96)
Chol (*N* = 148)	143.3 (24.86)		140.2 (25.02)	147.0 (27.76)
LDL (*N* = 148)	100.6 (25.08)		108.5 (29.26)	112.2 (33.07)
HDL (*N* = 148)	47.9 (10.92)		48.4 (11.46)	46.5 (11.07)
**AHT Variables after Segmentation and Coding**				
**Variable**	**Levels**	**% (N)-T1**	**% (N)-T2**	**% (N)-T3**
ATH_SBP (*N* = 148)	(0) <140 mmHg	73.0 (108)	64.2 (95)	54.7 (81)
	(1) ≥140 mmHg	27.0 (40)	35.8 (53)	45.3 (67)
ATH_DBP (*N* = 148)	(0) <90 mmHg	82.4 (122)	67.6 (100)	58.8 (87)
	(1) ≥90 mmHg	17.6 (26)	32.4 (48)	41.2 (61)

Note: Hb1Ac = glycosylated haemoglobin, AHT_SBP = systolic blood pressure, AHT_DBP = diastolic blood pressure, HR = heart rate, BMI = body mass index, Tri = triglycerides, Chol = cholesterol, LDL = low-density lipoprotein, HDL = high-density lipoprotein.

**Table 2:** Model used to predict systolic and diastolic AHT level at T2.

Prediction for Systolic AHT Level at T2							
Subscale	B	SD	Z	*p*	OR	CI for 95% OR	
						Lower	Upper
Constant	−7.06	1.88	−3.75	<0.001			
HR	0.03	0.02	1.77	0.076	1.03	0.99	1.06
BMI	0.16	0.05	2.96	0.003	1.17	1.05	1.31
**Prediction for Diastolic AHT Level at T2**							
**Subscale**	**B**	**SD**	**Z**	***p***	**OR**	**CI for 95% OR**	
						**Lower**	**Upper**
Constant	−10.17	2.17	−4.69	<0.001			
${\left(Sex\right)}_{1}$	−0.69	0.40	−1.73	0.084	0.50	0.23	1.09
HR	0.06	0.02	3.24	0.001	1.06	1.02	1.11
BMI	0.14	0.06	2.29	0.022	1.15	1.02	1.31
Chol	0.01	0.01	1.56	0.118	1.01	1.00	1.03

Note: Sex = sex, HR = heart rate, BMI = body mass index, Chol = cholesterol, B = parameter estimated, SD = standard deviation, Z = Z statistic, *p* = *p*-value, OR = odds ratio, CI = confidence interval, Lower = lower limit of the CI, Upper = upper limit of the CI.

**Table 3:** Model prediction of systolic and diastolic AHT at T3.

Prediction for Systolic AHT Level at T3							
Subscale	B	SD	Z	*p*	OR	CI for 95% OR	
						Lower	Upper
Constant	−7.35	1.94	−3.79	<0.001			
HR	0.03	0.02	1.53	0.127	1.03	0.99	1.06
BMI	0.11	0.06	1.79	0.074	1.17	0.99	1.27
Tri	0.02	0.01	2.00	0.045	1.02	1.00	1.03
**Prediction for Diastolic AHT** **Level at T3**							
**Subscale**	**B**	**SD**	**Z**	***p***	**OR**	**CI for 95% OR**	
						**Lower**	**Upper**
Constant	−4.74	1.34	−3.51	<0.001			
BMI	0.17	0.05	3.29	0.001	1.17	0.99	1.27

Note: HR = heart rate, BMI = body mass index, Tri = triglycerides, B = estimated parameter, SD = standard deviation.

## Data Availability

Data available under request to the corresponding author.

## References

[1] Mostaza J.M., Pintó X., Armario P., Masana L., Real J.T., Valdivielso P., Arrobas-Velilla T., Baeza-Trinidad R., Calmarza P., Cebollada J. (2022). SEA 2022 Standards for Global Control of Cardiovascular Risk. Clin. Investig. Arterioscler..

[2] Arnett D.K., Blumenthal R.S., Albert M.A., Buroker A.B., Goldberger Z.D., Hahn E.J., Himmelfarb C.D., Khera A., Lloyd-Jones D., McEvoy J.W. (2019). 2019 ACC/AHA Guideline on the Primary Prevention of Cardiovascular Disease: A Report of the American College of Cardiology/American Heart Association Task Force on Clinical Practice Guidelines. Circulation.

[3] Gorostidi M., Gijón-Conde T., De la Sierra A., Rodilla E., Rubio E., Vinyoles E., Oliveras A., Santamaría R., Segura J., Molinero A. (2022). Guía práctica sobre el diagnóstico y tratamiento de la hipertensión arterial en España, 2022. Sociedad Española de Hipertensión–Liga Española para la Lucha contra la Hipertensión Arterial (SEH-LELHA). Hipertens. Riesgo Vasc..

[4] Quesada-Caballero M., Carmona-García A., Chami-Peña S., Albendín-García L., Membrive-Jiménez C., Romero-Béjar J.L., la Fuente G.A.C.-D. (2023). COVID-19 and the Use of Angiotensin II Receptor Blockers in Older Chronic Hypertensive Patients: Systematic Review and Meta-Analysis. Medicina.

[5] Yang Y.-S., Pei Y.-H., Gu Y.-Y., Zhu J.-F., Yu P., Chen X.-H. (2023). Association between short-term exposure to ambient air pollution and heart failure: An updated systematic review and meta-analysis of more than 7 million participants. Front. Public Health.

[6] Gordon J., Miller G.C., Britt H. (2018). What are chronic conditions that contribute to multimorbidity?. Aust. J. Gen. Pract..

[7] Unger T., Borghi C., Charchar F., Khan N.A., Poulter N.R., Prabhakaran D., Ramirez A., Schlaich M., Stergiou G.S., Tomaszewski M. (2020). 2020 International Society of Hypertension Global Hypertension Practice Guidelines. Hypertension.

[8] Ott C., Schmieder R.E. (2022). Diagnosis and treatment of arterial hypertension 2021. Kidney Int..

[9] Adler A., Agodoa L., Algra A., Asselbergs F.W., Beckett N.S., Berge E., Black H., Brouwers F.P.J., Brown M., Bulpitt C.J. (2021). Pharmacological blood pressure lowering for primary and secondary prevention of cardiovascular disease across different levels of blood pressure: An individual participant-level data meta-analysis. Lancet.

[10] Benetos A., Petrovic M., Strandberg T. (2019). Hypertension Management in Older and Frail Older Patients. Circ. Res..

[11] Carey R.M., Wright J.T., Taler S.J., Whelton P.K. (2021). Guideline-Driven Management of Hypertension: An Evidence-Based Update. Circ. Res..

[12] Ajabnoor G.M.A., Bahijri S., Alamoudi A.A., Al Raddadi R., Al-Ahmadi J., Jambi H., Borai A., Toumilehto J. (2021). The association between hypertension and other cardiovascular risk factors among non-diabetic Saudis adults-A cross sectional study. PLoS ONE.

[13] Wang D., Hu B., Hu C., Zhu F., Liu X., Zhang J., Wang B., Xiang H., Cheng Z., Xiong Y. (2020). Clinical Characteristics of 138 Hospitalized Patients with 2019 Novel Coronavirus-Infected Pneumonia in Wuhan, China. JAMA.

[14] CDC How to Protect Yourself and Others. https://www.cdc.gov/coronavirus/2019-ncov/prevent-getting-sick/prevention.html.

[15] Guillem F.C. (2020). Opportunities and threats for prevention and health promotion and the PAPPS in the context of the COVID-19 pandemic. Aten. Primaria.

[16] Quesada-Caballero M., Carmona-García A., Chami-Peña S., Caballero-Mateos A.M., Fernández-Martín O., Cañadas-De la Fuente G.A., Romero-Bejar J.L. (2023). Telemedicine in Elderly Hypertensive and Patients with Chronic Diseases during the COVID-19 Pandemic: A Systematic Review and Meta-Analysis. J. Clin. Med..

[17] Paice J.A. (2022). Cancer pain during an epidemic and a pandemic. Curr. Opin. Support. Palliat. Care.

[18] García-Lara R.A., Suleiman-Martos N., Membrive-Jiménez M.J., García-Morales V., Quesada-Caballero M., Guisado-Requena I.M., Gómez-Urquiza J.L. (2022). Prevalence of Depression and Related Factors among Patients with Chronic Disease during the COVID-19 Pandemic: A Systematic Review and Meta-Analysis. Diagnostics.

[19] Agresti A. (2015). Foundations of Linear and Generalized Linear Models.

[20] World Medical Association (2013). World Medical Association Declaration of Helsinki: Ethical principles for medical research involving human subjects. JAMA.

[21] Chaput J.-P., Willumsen J., Bull F., Chou R., Ekelund U., Firth J., Jago R., Ortega F.B., Katzmarzyk P.T. (2020). 2020 WHO guidelines on physical activity and sedentary behaviour for children and adolescents aged 5–17 years: Summary of the evidence. Int. J. Behav. Nutr. Phys. Act..

[22] Eckstrom E., Neukam S., Kalin L., Wright J. (2020). Physical Activity and Healthy Aging. Clin. Geriatr. Med..

[23] Bellettiere J., LaMonte M.J., Evenson K.R., Rillamas-Sun E., Kerr J., Lee I.-M., Di C., Rosenberg D.E., Stefanick M.L., Buchner D.M. (2019). Sedentary Behavior and Cardiovascular Disease in Older Women: The OPACH Study. Circulation.

[24] Hermelink R., Leitzmann M.F., Markozannes G., Tsilidis K., Pukrop T., Berger F., Baurecht H., Jochem C. (2022). Sedentary behavior and cancer–an umbrella review and meta-analysis. Eur. J. Epidemiol..

[25] Ekelund U., Tarp J., Steene-Johannessen J., Hansen B.H., Jefferis B., Fagerland M.W., Whincup P., Diaz K.M., Hooker S.P., Chernofsky A. (2019). Dose-response associations between accelerometry measured physical activity and sedentary time and all cause mortality: Systematic review and harmonised meta-analysis. BMJ.

[26] NHS Physical Activity Guidelines for Older Adults. https://www.nhs.uk/live-well/exercise/exercise-guidelines/physical-activity-guidelines-older-adults/.

[27] CDC How Much Physical Activity do Older Adults Need? Physical Activity. https://www.cdc.gov/physicalactivity/basics/older_adults/index.htm.

[28] Estruch R., Ros E., Salas-Salvadó J., Covas M.-I., Corella D., Arós F., Gómez-Gracia E., Ruiz-Gutiérrez V., Fiol M., Lapetra J. (2018). Primary Prevention of Cardiovascular Disease with a Mediterranean Diet Supplemented with Extra-Virgin Olive Oil or Nuts. N. Engl. J. Med..

[29] Gotanda H., Liyanage-Don N., Moran A.E., Krousel-Wood M., Green J.B., Zhang Y., Nuckols T.K. (2022). Changes in Blood Pressure Outcomes Among Hypertensive Individuals During the COVID-19 Pandemic: A Time Series Analysis in Three US Healthcare Organizations. Hypertension.

[30] Nozato Y., Yamamoto K., Rakugi H. (2023). Hypertension management before and under the COVID-19 pandemic: Lessons and future directions. Hypertens. Res..

